# Use of the urine Determine LAM test in the context of tuberculosis diagnosis among inpatients with HIV in Ghana: a mixed methods study

**DOI:** 10.3389/fpubh.2023.1271763

**Published:** 2024-01-05

**Authors:** Johanna Åhsberg, Britt Pinkowski Tersbøl, Peter Puplampu, Augustine Kwashie, Joseph Oliver Commey, Yaw Adusi-Poku, Ellen Moseholm, Åse Bengård Andersen, Ernest Kenu, Margaret Lartey, Isik Somuncu Johansen, Stephanie Bjerrum

**Affiliations:** ^1^Research Center of Infectious Diseases, Department of Clinical Research, University of Southern Denmark, Odense, Denmark; ^2^Mycobacterial Centre for Research Southern Denmark, MyCRESD, Department of Infectious Diseases, Odense University Hospital Odense, Odense, Denmark; ^3^International Reference Laboratory of Mycobacteriology, Statens Serum Institut, Copenhagen, Denmark; ^4^Global Health Section, Department of Public Health, University of Copenhagen, Copenhagen, Denmark; ^5^Department of Medicine and Therapeutics, Medical School, College of Health Sciences, University of Ghana, Accra, Ghana; ^6^Department of Medicine, Tema General Hospital, Tema, Ghana; ^7^Department of Medicine, Lekma Hospital, Teshie, Ghana; ^8^National Tuberculosis Control Programme, Ghana Health Service, Korle-Bu, Accra, Ghana; ^9^Department of Infectious Diseases, Copenhagen University Hospital, Hvidovre, Denmark; ^10^Department of Public Health, Faculty of Health and Medical Sciences, University of Copenhagen, Copenhagen, Denmark; ^11^Department of Infectious Diseases, Copenhagen University Hospital Rigshospitalet, Copenhagen, Denmark; ^12^Department of Epidemiology and Disease Control, University of Ghana, Accra, Ghana

**Keywords:** HIV, tuberculosis, diagnosis, Xpert MTB/RIF, Determine LAM, qualitative interviews, mixed methods

## Abstract

**Background:**

The urine Determine LAM test has the potential to identify tuberculosis (TB) and reduce early mortality among people living with HIV. However, implementation of the test in practice has been slow. We aimed to understand how a Determine LAM intervention was received and worked in a Ghanaian in-hospital context.

**Design/Methods:**

Nested in a Determine LAM intervention study, we conducted a two-phase explanatory sequential mixed methods study at three hospitals in Ghana between January 2021 and January 2022. We performed a quantitative survey with 81 healthcare workers (HCWs), four qualitative focus-group discussions with 18 HCWs, and 15 in-depth HCW interviews. Integration was performed at the methods and analysis level. Descriptive analysis, qualitative directed content analysis, and mixed methods joint display were used.

**Results:**

The gap in access to TB testing when relying on sputum GeneXpert MTB/Rif alone was explained by difficulties in obtaining sputum samples and an in-hospital system that relies on relatives. The Determine LAM test procedure was experienced as easy, and most eligible patients received a test. HCWs expressed that immediate access to Determine LAM tests empowered them in rapid diagnosis. The HCW survey confirmed that bedside was the most common place for Determine LAM testing, but qualitative interviews with nurses revealed concerns about patient confidentiality when performing and disclosing the test results at the bedside. Less than half of Determine LAM-positive patients were initiated on TB treatment, and qualitative data identified a weak link in the communication of the Determine LAM results. Moreover, HCWs were reluctant to initiate Determine LAM-positive patients on TB treatment due to test specificity concerns. The Determine LAM intervention did not have an impact on the time to TB treatment as expected, but patients were, in general, initiated on TB treatment rapidly. We further identified a barrier to accessing TB treatment during weekends and that treatment by tradition is administrated early in the morning.

**Conclusion:**

The Determine LAM testing was feasible and empowered HCWs in the management of HIV-associated TB. Important gaps in routine care and Determine LAM-enhanced TB care were often explained by the context. These findings may inform in-hospital quality improvement work and scale-up of Determine LAM in similar settings.

## 1 Introduction

Tuberculosis (TB) diagnostic strategies that are non-sputum-based and available at the point-of-care (POC) are urgently needed. As the immune system deteriorates among people living with HIV (PWH), the diagnosis of TB is challenged by presentation with severe disease, sputum-scarce pulmonary TB (1, 2), extrapulmonary TB (EPTB), and disseminated TB. Sputum Xpert MTB/RIF or Xpert MTB/RIF Ultra assay (hereafter “Xpert,” Cepheid, Sunnyvale, California, United States) is recommended as a first-line test to assess for TB among PWH (3). Since 2015, WHO has recommended the use of urine lateral flow lipoarabinomannan assay, Determine™ TB LAM Ag assay (Determine LAM; Abbott, Chicago, IL, USA), a simple POC test, to assist TB diagnosis among PWH (4). Determine LAM is commercially available, detects all forms of TB, and provides a result after 25 min (5). It has the potential to assist timely HIV-associated TB diagnosis and TB treatment initiation (1, 6, 7), and two randomized controlled trials found that POC use of Determine LAM can impact early mortality among PWH inpatients (2, 8). Nevertheless, the scale-up of Determine LAM has been slow. A survey from countries with a high incidence of HIV-associated TB identified uptake of Determine LAM to be challenged by national regulatory agency test approval procedures, limited TB budgets, and lack of local test implementation data (9). A qualitative study assessing users’ perspectives of Determine LAM highlighted the WHO conditional Determine LAM eligibility criteria (10) and was concerned with test specificity and interpretation of test results as possible barriers to test adoption and use (10, 11). However, in-depth knowledge of contextual facilitators and barriers to Determine LAM implementation in real-world practice is limited. Such contextual factors are often neglected in randomized controlled trials (12) but are important to understand when new TB diagnostic strategies are implemented (13, 14).

In a multi-center stepped wedge cluster-randomized Determine LAM intervention study (TBPOC study), the Determine LAM test was made available for use among severely ill inpatients with HIV admitted at three hospitals in Ghana (15). The intervention was strengthened by training and performance feedback among the healthcare workers (HCWs) involved in patient care at the study sites. In the Determine LAM intervention study, patients were severely immunosuppressed with a high rate of TB symptoms, but less than half of patients had a routine sputum-based test for TB. When the Determine LAM test was made available, the TB diagnostic yield and the probability of TB treatment initiation increased (15). However, while test uptake among HCWs was high, only half of Determine LAM-positive patients were initiated TB treatment.

This mixed methods study seeks to better understand how the Determine LAM test intervention was received and worked in a Ghanaian in-hospital context.

## 2 Materials and methods

### 2.1 Study design

**Figure 1:**
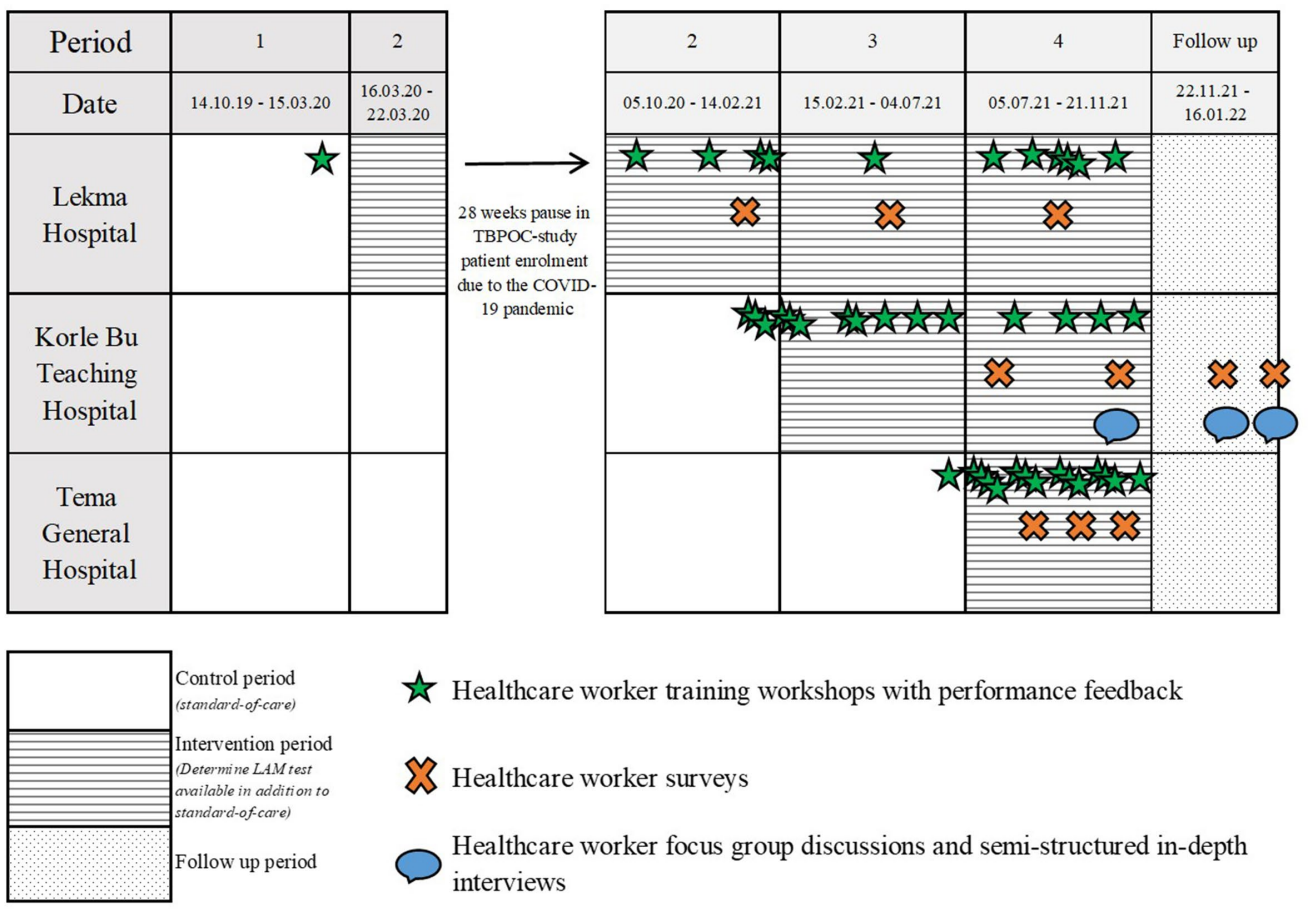
The mixed methods study nested into the stepped wedge cluster-randomized trial implementing the Determine LAM test at three hospitals in Ghana.

**Figure 2:**
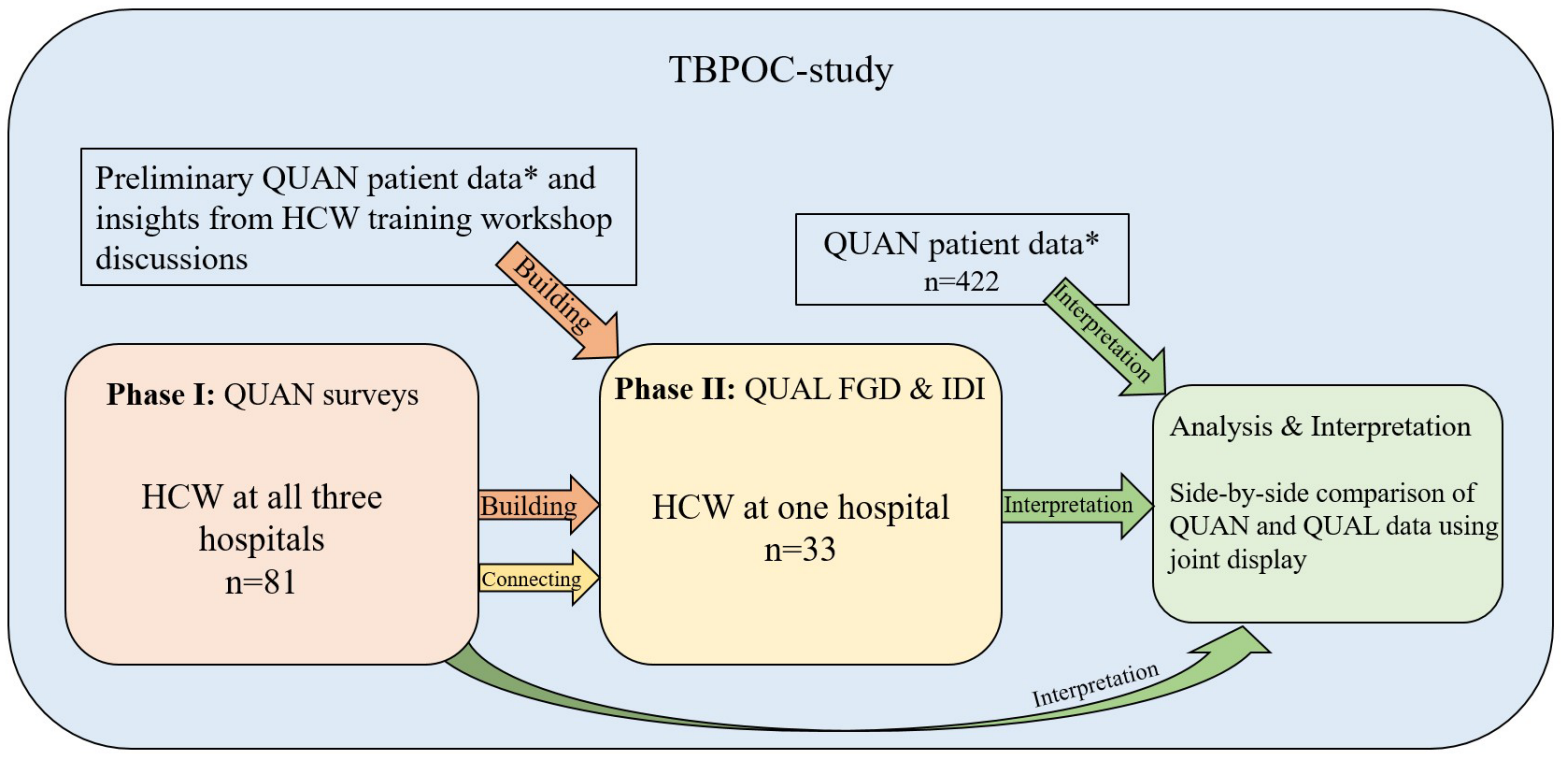
An overview of the explanatory sequential mixed methods study. QUAN, quantitative data; QUAL, qualitative data; HCW, healthcare worker; FGD, focus group discussions; IDI, Semi-structured in-depth interviews. *QUAN patient data are key findings from the Determine LAM intervention study reflecting Determine LAM study patient and process outcomes.

This is a two-phase, explanatory sequential mixed methods study (16, 17) using an approach where the HCW survey, qualitative in-depth interviews (IDIs), and focus group discussions (FGDs) were nested into the TBPOC study (Figure 1). As described in detail before, the TBPOC study is an open-label multi-center stepped wedge cluster-randomized trial evaluating the impact of the Determine LAM test on timely TB investigation, diagnosis, and treatment among severely ill PHW on admission^1^ (15). In the first phase, an HCW survey was performed at three hospitals between January 2021 and January 2022 to address the Determine LAM feasibility and ease of use. The second phase was conducted at one of the hospitals between October 2021 and January 2022 and included the collection of qualitative data from FGDs and IDIs to provide an in-depth understanding of the user perspectives and the context for Determine LAM uptake and use. Data integration of the TBPOC study (15, 18), HCW survey, and the FGD and IDI occurred at the methods level and the analysis and interpretation level (Figure 2; Table 1).

**Table 1:** Methods and participants overall and by hospital in the mixed methods study nested in the TBPOC study implementing the Determine LAM test.

Methods	Data type	Total (all study sites)	Lekma Hospital	Korle Bu Teaching Hospital	Tema General Hospital
Training workshops (TBPOC study) Key points from discussions with healthcare workers were used in the *building* of the qualitative topic guides.	QUAN	694 healthcare workers^a^/ 42 sessions	137 healthcare workers^a^/ 12 sessions	385 healthcare workers^a^/ 15 sessions	172 healthcare workers^a^/ 15 sessions
Patient data (TBPOC study) Preliminary TBPOC study data (patient and process data) was used in the *building* of the qualitative topic guides. Key findings from the TBPOC study were used as pre-defined codes in the qualitative directed content analysis and in the mixed methods joint display.	QUAN	422 patients/ 1 trial	91 patients/ 1 trial	142 patients/ 1 trial	189 patients/ 1 trial
HCW survey Preliminary healthcare worker survey results were used in the *building* of the qualitative topic guides, and to inform purposive sampling of participants to the qualitative FGD and IDI through *connecting*. Key findings from the survey were used as pre-defined codes in the qualitative directed content analysis and in the mixed methods joint display.	QUAN	81 healthcare workers/ 81 surveys	25 healthcare workers/ 25 surveys	44 healthcare workers/ 44 surveys	12 healthcare workers/ 12 surveys
FGD Summaries and transcripts were analyzed in the *qualitative-directed content analysis*. Quotations from the content analysis were arrayed against key quantitative findings in a mixed methods joint display.	QUAL	18 healthcare workers/ 4 focus group discussions	b	18 healthcare workers/ 4 focus group discussions	b
IDI Summaries and transcripts were analyzed in the qualitative-directed content analysis. Quotations from the content analysis were arrayed against key quantitative findings in a mixed methods joint display.	QUAL	15 healthcare workers/ 15 in-depth interviews	b	15 healthcare workers/ 15 in-depth interviews	b

Determine LAM, lateral flow urine lipoarabinomannan assay, Determine™ TB LAM Ag test (Abbott Laboratories, Chicago, IL, USA); QUAN, quantitative; QUAL, qualitative; FGD, focus group discussions; IDI, semi-structured in-depth interview. a, The same participant may have participated in one or more sessions within the same hospital. b, FGD and IDI were only conducted at Korle Bu Teaching Hospital.

### 2.2 Study sites and context for Determine LAM test implementation

The study sites and the routine TB diagnostic care cascade are described previously (15, 18). Sites include Lekma Hospital (LH), Tema General Hospital (TGH), and Korle Bu Teaching Hospital (KBTH), all major hospitals with mainly an urban population uptake. HIV and TB care facilities are available with diagnosis and treatment provided free of charge to the patients. As part of the TBPOC study, the Determine LAM test was made available at the medical wards for TB diagnosis among newly admitted PWH if severely ill, with advanced HIV or a positive WHO 4-symptom screen (W4SS) (15). Request and use of the Determine LAM tests were at the discretion of the treating HCWs. The tests were placed conveniently at the wards with all required test materials, guidelines for using Determine LAM (4, 19), and the manufacturer’s instructions (20). The introduction of Determine LAM at the hospitals was strengthened by monthly training of HCWs comprising performance feedback, review of the guidelines for the diagnosis of HIV-associated TB (21), and training in the theoretical and practical use of Determine LAM (4, 19, 20). A total of 42 formal training sessions led by the first author (J.Å.) in collaboration with hospital management at the three study sites and the National Tuberculosis Control Program in Ghana were held.

Simplified, the Determine LAM test procedure follows four steps: 1. Remove the foil on the test strip; 2. Add 60 μL urine to the pad; 3. Wait 25 min; and 4. Read the result using the reference scale card (invalid, negative, or grade 1–4 positive) (19).

### 2.3 Study population and data collection

#### 2.3.1 Quantitative healthcare worker survey

In conjunction with the training sessions, HCWs at the three hospitals were approached purposefully and were based on convenience to fill out a study-specific pre-tested paper questionnaire on the Determine LAM use. Overall topics included HCW characteristics, workload, and preferred location for Determine LAM testing, Determine LAM use and interpretation, and urine sampling. In total, 81 HCWs completed the survey.

#### 2.3.2 Qualitative FGDs and IDIs

We conducted FGDs to explore group dynamics and contrast views on the discussed topics, while IDIs were conducted to get a deeper understanding of the individual HCW opinions and perspectives. For both FGDs and IDIs, we used purposive sampling and approached HCWs based on convenience to ensure representation of different genders, staff categories, experience, and departments (internal medicine, infectious diseases, and respiratory medicine, incl. COVID-19 units). All participants had completed the HCW survey before the qualitative data collection. To facilitate a free-flowing and open FGD for all participants, HCWs were grouped according to work role and experience.

Two semi-structured topic guides were built for the FGDs and IDIs, respectively (Supplementary Table S1), based on insights gained from the HCW training and the HCW survey and questions raised during the TBPOC study. The FGDs and IDIs were conducted at KBTH in English, the official language in Ghana, and the language used by HCWs in their daily work. Sessions lasted between 40 and 60 min. They were moderated by the first author (J.Å.), who is a medical doctor with knowledge of HIV and TB, qualitative research, and who had resided and worked at the study sites as a clinical researcher for 2.5 years at the time of the qualitative interviews. A Ghanaian research assistant with insight into the local context and several local languages participated during the interviews to avoid misunderstandings.

Data saturation occurred after four FGDs totaling 18 participants (four TB diagnostic HCWs, four junior doctors, five junior nurses, and five senior nurses) and 15 IDIs (eight nurses, public health nurses or counselors, and seven doctors). Of the 39 HCWs invited to either an FGD or an IDI, six declined because they did not want to participate (three), were too busy (two), or were sick (one). After each FGD or IDI, the main findings were summarized. All FGDs and IDIs were audio-recorded and transcribed verbatim and digitally stored in a pseudo-anonymized form at OPEN, Open Patient data Explorative Network, Odense University Hospital.

### 2.4 Data analysis

#### 2.4.1 Quantitative analysis

For the HCW survey analysis, we used descriptive statistics to present frequencies and percentages, medians with interquartile ranges, and frequencies and percentages using STATA 17.0 software (StataCorp. 2021 Stata Statistical Software: Release 17. College Station, TX: StataCorp LLC).

#### 2.4.2 Qualitative analysis

Directed qualitative content analysis was used (22, 23). First, we (J.Å., B.P.T., and S.B.) developed a code scheme based on the research question, the topic guides, key preliminary findings from the TBPOC study (15, 18) and the HCW survey, and key findings from published material in the field (9–11). Additionally, we included a few broad codes, which are as follows: What dilemmas do HCWs/patients experience? Does power or lack of power manifest itself in the material? and What is seen to be a priority among HCWs? The FGD and IDI summaries and the transcripts were analyzed for pre-determined codes using an initially deductive approach (24). When the material did not relate to any pre-defined code, we used an abductive approach to identify new codes, and the code scheme was revised and refined (22). Meaning-units in the form of representative quotes were continuously registered into the code scheme. When going back and forth between different codes with representative meaning units, summaries, and transcripts, abstraction of themes and over-arching themes emerged (23).

#### 2.4.3 Integration

Integration at the methods level occurred when preliminary results from the TBPOC study (15, 18) and the HCW survey contributed to *building* the qualitative topic guides and when the HCW survey was *connected* to the qualitative data sampling (Figure 2) (25). Integration at the analysis and interpretation level occurred through joint display analysis and in the discussion (25, 26). Importantly, by arraying quotes from the comprehensive qualitative content analysis code scheme against key quantitative findings, the data were explicitly brought together in a side-by-side comparison to assess how the qualitative findings could explain the quantitative results (25–27).

## 3 Results

### 3.1 Quantitative results

The TBPOC study results have been published elsewhere, including the in-hospital TB diagnostic care cascade (18) and outcomes of the Determine LAM intervention study (15). In summary, we found that less than half of severely ill inpatients with HIV obtained a sputum Xpert for assessment of TB during admission. During the implementation of the Determine LAM intervention, 162 of the 174 (93.1%) patients were tested with Determine LAM, the majority bedside, but only 19 of the 41 (46.3%) Determine LAM positive patients were initiated on TB treatment. Implementation of Determine LAM increased TB diagnosis and reduced time to TB diagnosis but did not affect the time to TB treatment initiation.

Of the 81 respondents to the HCW survey, 42 (51.9%) were nurses, 28 (34.6%) were doctors, and 11 (13.6%) were from other staff categories. Among all, 30 (37%) HCWs had more than 5 years of work experience. Of the 80 respondents, 37 (46.3%) tested 0–1 patients with Determine LAM each week, 28 (35%) tested two or more patients with Determine LAM each week, and 15 (18.8%) did not have access to the Determine LAM, e.g., worked in a laboratory. For a detailed description of HCW characteristics, see Supplementary Table S2. Overall, 68/69 (98.6%) respondents experienced the Determine LAM procedure as easy or very easy (Table 2). The majority, 60/69 (87.0%), estimated that they spent 10 min or less on informing the patient about the Determine LAM test and a median of 30 (IQR 25–40) min to complete the Determine LAM test per patient. Among respondents, 56/69 (81.2%) stated that a urine sample could be obtained within 60 min, with 11/69 (16.4%) finding it difficult or very difficult to obtain the urine sample.

**Table 2:** Results from the quantitative healthcare worker survey on Determine LAM feasibility and ease of use, overall and stratified by hospital, *n* = 81.

Workload and location for Determine LAM testing		Overall population (*n* = 81)	LH (*n* = 25)	KBTH (*n* = 44)	TGH (*n* = 12)
Do you perform the Determine LAM yourself? *n* = 77	Yes or Sometimes	40 (51.9)	15 (60.0)	15 (37.5)	10 (83.3)
	No	37 (48.1)	10 (40.0)	25 (62.5)	2 (16.7)
How long time do you spend on average to inform the patients about Determine LAM testing? *n* = 69	0–5 min	34 (49.3)	9 (40.9)	19 (54.3)	6 (50.0)
	5–10 min	26 (37.7)	10 (45.5)	11 (31.4)	5 (41.7)
	> 10 min	9 (13.0)	3 (13.6)	5 (14.3)	1 (8.33)
How long time is required on average before urine is obtained from the patient, counting from the time you requested it? *n* = 69	0–10 min	18 (26.1)	6 (27.3)	9 (25.7)	3 (25.0)
	10–30 min	22 (31.9)	7 (31.8)	10 (28.6)	5 (41.7)
	30–60 min	16 (23.2)	8 (36.4)	6 (17.1)	2 (16.7)
	>60 min	11 (15.9)	1 (4.6)	8 (22.9)	2 (16.7)
	Often not possible within the same day	2 (2.9)	0 (0)	2 (5.7)	0 (0.0)
How long do you spend on average for the total Determine LAM activity per patient? *n* = 61, median minutes (IQR)		30 (25–40)	30 (25–40)	30 (20–35)	32.5 (28.8–52.5)
Where do you, in general, perform the Determine LAM test? *n* = 69 (more than one alternative allowed per respondent)	Bedside	60 (87.0)	23 (92.0)	27 (84.4)	10 (83.3)
	Consultation room	1 (1.4)	0 (0.0)	1 (3.1)	0 (0.0)
	Designated room at the ward	3 (4.3)	1 (4.0)	3 (9.4)	0 (0.0)
	Designated room outside the ward	1 (1.4)	0 (0.0)	1 (3.1)	0 (0.0)
	Laboratory	5 (7.2)	1 (4.0)	2 (6.3)	2 (16.7)
	Other, describe	3 (4.3)	0 (0.0)	3 (9.4)	0 (0.0)
If otherwise, provide a description of where the Determine LAM test was performed.	Designated room at consulting area; In the washroom; At the out-patient clinic when necessary				
What would to you be the optimal location to perform the Determine LAM test? *n* = 75 (more than one alternative allowed per respondent)	Bedside	35 (46.7)	16 (64.0)	14 (36.8)	5 (41.7)
	Consultation room	8 (10.7)	1 (4.0)	6 (15.8)	1 (8.3)
	Designated room at the ward	21 (28.0)	5 (20.0)	15 (39.5)	1 (8.3)
	Laboratory	20 (26.7)	11 (44.0)	4 (10.5)	5 (41.7)
	Other, describe	1 (1.3)	0 (0.0)	1 (2.6)	0 (0.0)
If otherwise, provide a description of the optimal place for the Determine LAM test to be performed.	At the OPD, when the patient is clinically suspected of TB.				

Determine LAM use and interpretation		Overall population (*n* = 81)	LH (*n* = 25)	KBTH (*n* = 44)	TGH (*n* = 12)
How do you experience the Determine LAM info leaflet? *n* = 70	Very easy	28 (40)	16 (66.7)	9 (25.7)	3 (27.3)
	Easy	40 (57.1)	8 (33.3)	24 (68.6)	8 (72.7)
	Difficult	2 (2.9)	0 (0.0)	2 (5.7)	0 (0.0)
	Very difficult	0 (0.0)	0 (0.0)	0 (0.0)	0 (0.0)
How do you experience the Determine LAM procedure overall? *n* = 69	Very easy	27 (39.1)	16 (66.7)	9 (27.3)	2 (16.7)
	Easy	41 (59.4)	8 (33.3)	23 (69.7)	10 (83.3)
	Difficult	1 (1.5)	0 (0.0)	1 (3.0)	0 (0.0)
	Very difficult	0 (0.0)	0 (0.0)	0 (0.0)	0 (0.0)
Did you use the reference scale card to judge if Determine LAM test was positive or negative? *n* = 65	Yes	58 (89.2)	19 (82.6)	27 (90.0)	12 (100.0)
	No	7 (10.8)	4 (17.4)	3 (10.0)	0 (0.0)
Did you use the reference scale card to grade the Determine LAM band intensity? *n* = 66	Yes	57 (86.4)	19 (82.6)	26 (83.9)	12 (100.0)
	No	9 (13.6)	4 (17.4)	5 (16.1)	0 (0.0)
How did you experience interpretation of the Determine LAM test results with discrimination between the 4 intensity bands? *n* = 62	Very easy	19 (30.6)	11 (52.4)	3 (10.3)	5 (41.7)
	Easy	36 (58.1)	9 (42.9)	20 (69.0)	7 (58.3)
	Difficult	5 (8.1)	1 (4.8)	4 (13.8)	0 (0.0)
	Very difficult, describe	2 (3.2)	0 (0.0)	2 (3.2)	0 (0.0)
If very difficult to discriminate between the 4 intensity bands, describe:	At the first encounter but upon subsequently explaining the procedure was ok; Sometimes it is difficult to see the trace line.				

Urine sample		Overall population (*n* = 81)	LH (*n* = 25)	KBTH (*n* = 44)	TGH (*n* = 12)
How do you experience obtaining urine samples from patient? *n* = 67	Very easy	17 (25.4)	9 (39.1)	4 (11.8)	4 (40.0)
	Easy	39 (58.2)	10 (43.5)	26 (76.5)	3 (30.0)
	Difficult	10 (14.9)	4 (17.4)	3 (8.8)	3 (30.0)
	Very difficult	1 (1.5)	0 (0.0)	1 (2.9)	0 (0.0)
How do you experience handling the urine sample? *n* = 68	Very easy	21 (30.9)	13 (54.2)	7 (20.6)	1 (10.0)
	Easy	47 (69.1)	11 (45.8)	27 (79.4)	9 (90.0)
	Difficult	0 (0.0)	0 (0.0)	0 (0.0)	0 (0.0)
	Very difficult	0 (0.0)	0 (0.0)	0 (0.0)	0 (0.0)
How do you experience disposal of the urine sample? *n* = 67	Very easy	24 (35.8)	13 (54.2)	9 (27.3)	2 (20.0)
	Easy	41 (61.2)	10 (41.7)	23 (69.7)	8 (80.0)
	Difficult	1 (1.5)	0 (0.0)	1 (3.0)	0 (0.0)
	Very difficult	1 (1.5)	1 (4.2)	0 (0.0)	0 (0.0)

LH, Lekma Hospital; KBTH, Korle Bu Teaching Hospital; TGH, Tema General Hospital; TB, Tuberculosis; Determine LAM, lateral flow urine lipoarabinomannan assay, Determine™ TB LAM Ag test (Abbott Laboratories, Chicago, IL, USA); NA, Not applicable. Data are *n* (%) unless stated otherwise.

While 60 of the 69 (87.0%) HCWs performed the Determine LAM test at the bedside, only 35/75 (46.7%) thought that the bedside was the optimal location for testing. Of the two largest HCW categories, 15 of the 26 (57.7%) doctors and 17 of the 40 (42.5%) nurses thought the bedside was the optimal place for Determine LAM testing.

Of the 65 HCWs, 7 (10.8%) did not use the reference scale card to interpret test results, and 7 of 62 (11.3%) thought that discrimination between the four intensity bands was difficult or very difficult.

### 3.2 Qualitative findings

Based on FGDs and IDIs, five overarching qualitative themes emerged.

#### 3.2.1 Socio-economic vulnerable patients receive sub-optimal management

HCWs highlighted that PWH often has limited financial resources and therefore cannot afford important diagnostic investigations for HIV-associated disease, e.g., costs for blood tests and out-of-pocket payments for chest X-rays.

*“However, to be honest, investigations are predominantly catered for by the relatives, like she said [another junior doctor]. So into much as an X-ray is required, they have to pay for it, but for the Xpert, that’s free, so it’s easier to get. The rate limiting step comes from the labs.”* FGD with an infectious diseases doctor.

Relatives played an important role both financially by covering medical costs and socially by running hospital errands, e.g., escorting the patient to chest X-ray or transporting the sputum sample to the laboratory and bringing back the test result to the ward. A nurse suggested that an in-hospital porter service would be helpful.

*“Yes, so you see, if relatives are not around, we have to wait for them to come before we even take the sample [sputum sample], that is the system that goes on here. But if you have a system that is available 24/7 even without the relatives, you can take the sample and that person takes it to the lab.”* IDI with a junior medical nurse.

It was a priority for HCWs that the Determine LAM test be offered free of charge to the patients and made accessible for use at the wards at their own discretion without relying on the patients*’* socio-economic situation.

#### 3.2.2 The Determine LAM test empowers you as a healthcare worker

Doctors found that the Determine LAM test facilitated TB diagnosis when used in addition to routine investigation. It was satisfactory to be able to initiate a TB investigation without depending on the laboratory. Nurses and doctors explained that the Determine LAM test would also increase patient motivation and satisfaction.

*“I have instances where a patient comes a week later and the results [sputum Xpert] are not yet in and we are still waiting. And it means probably we have to wait for another one week. That’s two weeks, when you can just do a bedside test. So I think it gives us the opportunity of early diagnosis for patients, reduces their illness time and to an extent, yes, it will help the patients to appreciate because now they come to the hospital with a problem, and they are going home with semblance of a solution rather than coming with a problem and going home with more questions.”* FGD with an infectious diseases doctor.

Doctors found the Determine LAM test especially useful for timely clinical decision-making among patients with severe immunosuppression, who are unable to produce sputum for Xpert, and patients with EPTB.

*“…but as I mentioned those with the pericardial effusion. Some of them that had abdominal Kochs [TB], those people were highly suppressed. They had like severe immunosuppression. They came as [Determine LAM test] positive. We did not waste time, we just started anti-Kochs [TB treatment”]*.” IDI with an infectious diseases doctor.

Furthermore, it was explained by several HCWs that many patients with presumptive TB are not able to produce a sputum sample, where urine for Determine LAM testing was easier to obtain.

*“If the patient is not able to produce the sputum, it’s one of the major things that we tend to experience. Some of the patients, you will ask them to cough and they bring out saliva instead of the sputum. So you are not able to do your diagnosis.” IDI with a* senior medical nurse.

#### 3.2.3 Bedside testing was preferred for POC testing but may violate patient confidentiality

Especially in the FGDs for junior and senior nurses, concerns were noted about patient confidentiality when performing bedside Determine LAM tests. Nurses thought that a dedicated room in the ward would be the optimal place for testing and disclosing the test results. To enhance patient confidentiality, mobile physical screens were used between patient beds. One nurse explained that to avoid co-patients listening in, test information and the actual procedure could be performed at a distance away from the beds.

*“When there is lack of space, you have no choice than to do it at the bedside. But you have to just screen [place physical screens between beds]…”* FGD with junior nurses.

It was explained that, in general, it was the nurses, counselors, or laboratory technicians who performed Determine LAM testing. However, when junior doctors discussed the optimal place for testing, they preferred POC testing bedside over laboratory testing to avoid delay and a mix-up of test results. They highlighted that the rapid nature of the test was a main strength, and performing it in centralized laboratories would be counter-intuitive.

An infectious diseases doctor was frustrated over the limited routine healthcare services at the hospital on the weekends and suggested that the Determine LAM test, together with CD4 cell count, should be accessible at all times and part of a POC package for patients with advanced HIV. Experienced difficulties initiating TB treatment on the weekends were highlighted.

*“…unfortunately a lot of things are under lock with a key. The unfortunate thing is Friday evening, Saturday, Sunday, we are the only people who exist in the hospital (laugh)” …*” *…waiting a whole three days and not knowing what to do, is not the best, you know. I mean even giving anti-Kochs [TB treatment], policy wise, Saturday, Sunday, I mean, they are not there, and it can be annoying.”* IDI with an infectious diseases doctor.

Several nurses highlighted the importance of performing the Determine LAM test in front of the patient to increase patient acceptance and, thereby, prognosis.

*“Then the patient can be informed at the same time, get better insight and attitude and will better accept the result. This would increase chance for a good outcome.”* IDI with a senior medical nurse.

One nurse suggested that HCW training on Determine LAM testing should include discussions on how to ensure patient confidentiality during bedside testing.

#### 3.2.4 The Determine LAM test use from a contextual perspective

Healthcare workers explained that more PWH would benefit from the test if it is available for use at the hospital point-of-entries (i.e., polyclinic, the emergency department, or the outpatient department) and possibly in the community.

*“…the community nurses will be able to get to them [PWH with likely TB] and do the necessary testing and provide the necessary services to them. Because yes we have severely immunosuppressed people who point-blank refuse to attend any health facility.”* FGD with senior nurses.

Delayed HIV screening was identified as a current barrier to Determine LAM use.

*“Because sometimes patients will be on the ward for perhaps a week or more before we think to do a retro screen [HIV test].”* IDI with a junior medical doctor.

Other suggested barriers for test use included material stock-out and limited access to CD4 cell counts.

*“Ahaa, so it becomes very problematic like programme wise we are not doing the CD4. If they [the patients] have to do it then they will have to pay. The CD4 with a full blood count, I think they are paying like 120 cedis.”* IDI with an infectious diseases doctor.

Finally, the HCWs highlighted the role of the Determine LAM test as an add-on TB test and feared that it, by mistake, could be perceived as a replacement for routine TB diagnostic tests such as the Xpert. Therefore, a few HCWs suggested emphasizing during training that the Determine LAM test result should be held up against clinical judgment and subsequent TB diagnostic investigation.

#### 3.2.5 Attitudes toward TB disease and impact on TB care

Several nurses expressed fear of becoming infected with TB when caring for patients and requested more training in TB transmission and how to safely provide TB care.

Healthcare workers noted that some patients did not want to get tested for TB out of fear or negative beliefs.

*“They get scared when they hear the word TB or HIV, yeah, they get scared. So just let them know, now TB is curable right? Yes, so you just let them know if the test turns out to be positive you can get cured. So they can feel at ease understanding take the test.”* FGD with junior nurses.

HIV testing and counseling was generally described as a successful practice, and nurses suggested a similar set-up when testing for TB in addition to HIV. Nurses believed it would increase the acceptance of the diagnosis. It was also highlighted that some patients need time to accept, and apart from taking help from a counselor, a trusted relative was sometimes helpful.

*“And some too they feel that when they get TB* [in addition to HIV]*, it is like their death warrant has been signed.”* IDI with a senior medical nurse.

### 3.3 Mixed methods results

The joint display resulted in seven themes based on content similarities (see Table 3).

**Table 3:** Arraying qualitative quotes with quantitative findings to better understand the Determine LAM test role in tuberculosis management among inpatients living with HIV in Ghana—a joint display.

Quantitative findings	Illustrative qualitative quotes	Themes with explanations of how the qualitative data contributed to a better understanding of the quantitative data
In the standard-of-care population of the TBPOC study, less than half (45.2%) of severely ill inpatients with HIV and high risk of TB obtained a routine test for TB (sputum Xpert).	*“For those who come in who are ill, who are admitted onto our wards we virtually would do a chest X-ray minimum for each of the patients and irrespective of what we are managing them for, because we know of the prevalence of HIV/TB co-infection, virtually everybody unless those who cannot produce sputum, everybody gets the opportunity to be investigated for TB.” Infectious diseases doctor*	Barriers to routine sputum Xpert testing Patient not being able to produce a sputum sample; Lack of relatives to assist in sample logistics; Negative beliefs about the disease
	*“So then for a LAM test, it can be performed right at the ward by the patient bedside, with immediate result so that I would not have to wait for relatives to first go and get me a sputum bottle, patients always produce urine, so getting a sample is far easier than in a patient who is not coughing and you would have to wait for them to get you a sputum sample.”* Junior medical doctor	
	*“There are certain patients, because of the stigma. I want to ask do you cough, and she see this red bottle, she would say no.”* TB diagnostic HCW	
In the HCW survey, almost all, 68/69 (98.6%), HCWs experienced the Determine LAM test procedure as easy or very easy. In the TBPOC study, 12/174 patients (6.8%) were not tested with the Determine LAM test despite being eligible. Reasons included disrupted communication between HCW (33.3%), the clinician wanting the infectious disease team to decide on testing (8.3%), the clinician not suspecting TB (8.3%), the patient being discharged before the test was requested (8.3%), the patient was dying (33.3%) or not willing to get tested (8.3%).	*“Because sometimes patients will be on the ward for perhaps a week or more before we think to do a retro screen [HIV test].”* Junior medical doctor	Barriers and facilitators to Determine LAM testing Barriers: Patients being unwilling to get tested (out of fear, or more trust in herbal medicine or believing the disease was spiritual); Delayed HIV screening Facilitators: Instant test, any trained HCW can test
	*“The challenges that we tend to have with the LAM test is that, we need to want it. The doctors need to embrace it, and it has to be prescribed for almost all of our patients who come on admission who we think have [HIV-associated] TB.”* Senior medical nurse	
	“…for instance with the sputum testing, they have to go and come back in 24 h for their result, and then a lot of people get frustrated. Some people will not even come back because they are like, why do I have to take transportation, go and come. But with the LAM, if it’s an instant thing, you cut down the costs for the patients, you cut down the chances of losing them to follow up…” Senior medical nurse	
	“I think that as a point-of-care test it can be done by any clinical staff with appropriate training, so the key word is training.” TB diagnostic staff	
In the TBPOC study it took a median of 4 days from hospital admission to have an Xpert result, and the Determine LAM intervention reduced the time from enrolment to TB diagnosis from a median of 2 days to a median of 0 days. More than 80% in the HCW survey estimated that urine could be obtained within 60 min, and that the overall Determine LAM activity per patient took a median of 30 min.	*“So those emergency cases that comes [in the weekend] and they are then suspected [of having TB], they find it difficult to take samples [for Xpert] until Monday.”* TB diagnostic HCW	Timely TB diagnosis when assisted by Determine LAM compared to using Xpert only Logistics with sputum sample transportation and pick up of results from the Xpert laboratory; Limited Xpert laboratory opening hours; Xpert cartridge stock-out; Technical issue with the Xpert machine
	*“…GeneXpert, the sputum, it takes like 100 min to a maximum two hours, but sometimes it takes days! We have to follow up to get the results, but this one it’s right by the bedside. You can do it and know the result. I think, it’s not to say that those [Xpert] are not important, but the point is, this [Determine LAM] will be a very helpful addition.”* Infectious diseases doctor	
	*There has been quite a number of instances where you send a patient from the consulting room to the X-ray room, and the machine is down or” dumsor,” power, there is no power. So, and even with the GeneXpert, the AFB, the DST, sometimes they do not have…ehh…materials [Xpert cartridges* etc.*] or sometimes their machine needs to be worked on. So then the 24 h I’m talking about extends to further notice, which is also an issue.” Senior medical nurse*	
	*“So I’m thinking that there is a lot of times where you tell somebody to produce sputum for testing, and they would tell you, ohh I cannot get it. But urine is something that everybody, as long as you are drinking water, you will pee. So it will be easier to get urine samples than other samples for testing.”* Senior medical nurse	
The majority, 142/162 (87.7%) of the Determine LAM tests in the TBPOC study were performed at the bedside. HCW in the HCW survey also reported that bedside was the routinely used place for testing in 87% of cases, but among respondents, more than half thought that a dedicated room at the ward or the laboratory would be the optimal place for testing. More doctors, 15/26 (57.7%), than nurses, 17/40 (42.5%), thought bedside was the optimal place for testing.	*“Even if you screen [use physical screens between beds]. At first you explain the procedure to the patient. The patient laying on the next bed can actually hear. And then they hear that…eei, you are about to perform tuberculosis test, then they get alarmed (laugh). So I still do not see it to be private enough.”* Senior medical nurse	The place of Determine LAM testing in clinical practice was at the bedside The observed difference between clinical practice and what HCW thought was the best place for Determine LAM testing was better understood in the qualitative data. Nurses were concerned about patient confidentiality, and to ensure patient confidentiality, they used physical screens between patient beds. Nurses thought that the optimal place for testing and disclosure of the result would be in a dedicated room in the ward, while one doctor explained that the Determine LAM test would lose its value as a point-of-care test if performed away from the patient.
	*“Most times we do it at the patient’s bedside but I think that we should have, not the lab per say, because we, nurses and doctors, can easily do it at the ward. We should have a special room for example the treatment room or procedure room, so when the sample is taken it is sent there for it to be done. So that patient privacy and disclosure of patient information will be maintained. Cause we talk about the patients’ rights. They have the right to privacy. When you do it, it should be done in an excluded environment so that when the result is positive, you will know the best way to disclose it to patient, not letting everybody know that the patient is positive. “*Senior medical nurse	
	*“It’s just that the aim of the test is to reduce the patient time, and get fast results. So if we take it to the lab., it means that it defeats the purpose of the test…” “…but it’s not a bad idea, it’s just not the best idea.”* Infectious diseases doctor	
In the HCW survey, around 10% of respondents did not use the reference scale card when Determine LAM testing, and more than 10% of respondents thought it was difficult to interpret the Determine LAM test results with discrimination between the four intensity bands	*“For the taking of the sample it is not anything difficult and then taking your test kit, dropping it onto it is not so much difficult. But I think where we need to dwell much is the interpretation of the result. The interpretation of the result is where I think we need to dwell much upon.”* Senior medical nurse	Determine LAM test interpretation Specifically, it was difficult to discriminate between grade 1 positive and negative. The risk of losing the small reference scale card was an experienced barrier to interpretation and may explain why some HCWs in the HCW did not use the card. Nurses and doctors explained that they took help from a colleague when they were in doubt about the test result.
	*“I think we did one, the line was very faint. I was finding it difficult to read whether it was positive or it was negative.”* Senior medical nurse	
	*“…at a point when I was explaining to one of the nurses at (ward name), the reference card that was given to them was nowhere to be found. So they did not like, I think they had put it somewhere, so they did not know where it was. Because it was in a folder somewhere on the desk.”* Junior medical doctor	
	*“…she wasn’t able to interpret on the numbers, the grading on the card, that was a challenge. She was not able to do it so well so I had to come in and explain to her how it is done.”* Senior medical nurse	
In the TBPOC study, 22/41 (53.7%) patients did not initiate TB treatment despite having a positive Determine LAM test with reasons including disrupted communication of result between HCW (13.6%), awaiting sputum Xpert (13.6%), being discharged without having a referral for treatment (9.1%), being referred to Chest clinic to initiate treatment but was not initiated (9.1%), another main diagnosis was more likely (18.2%), patient had comorbidities or was unstable (18.2%), the patient died before treatment was requested or the first dose was taken (9.1%) or nor reason registered (9.1%)	*“Ours [the nurses’ digital medical notes] is different and theirs [the doctors] is also different. So we cannot go into their notes. So with this one [the registration of the Determine LAM result], I think we will have a small sheet which is designed as maybe positive or negative, and we document it and then we will take it to the doctor to input it into their notes. I think that will be better.” Senior medical nurse*	Barriers to Determine LAM guided TB treatment initiation The new digital medical record was a potential weak link for bedside test result reporting. The dilemma with the initiation of TB treatment is knowing that the specificity is sub-optimal
	*“The LAM test is said not to be, so specific for mycobacterium tuberculosis right. And it may be positive with other mycobacterial species as well like with the mycobacterium avium complex and others. So it’s an issue with the specificity of the test as to whether it is really TB or something else.”* Junior medical doctor	
	*“Yes, so the issue of the [Determine LAM test] false positives makes it a little bit uncomfortable. Because, yeah like, did I make the right choice?”* Infectious diseases doctor	
The TBPOC study was not able to show any intervention impact on time to TB treatment initiation that remained at a median 3 days from enrolment. It was registered in the study that a few cases that were Determine LAM positive were not initiated on TB treatment as the clinical team wanted to await a sputum Xpert result.	*“…waiting a whole three days and not knowing what to do, is not the best, you know. I mean even giving anti-Kochs, policy wise, Saturday, Sunday, I mean, they are not there, and it can be annoying.”* Infectious diseases doctor	Routine healthcare practices compromised timely TB treatment initiation Awaiting needed investigation, e.g., liver function tests that were challenging to obtain due to cost; Limited access to TB treatment and patient registration in the chest clinic in the weekends; Limited access to public health nurses in the weekend to perform pre-counseling and supply the TB medication; TB medications are only given early mornings
	*“So some of them when they come in, you may have some challenges with even doing their labs. Because they are very sick and economically they have lost all their sustenance, okay. So it takes a while for us to get all their investigations to be able to take the final decision to start them on the TB medications.”* Infectious Diseases doctor	
	*“In fact, the duration is a bit long. Ideally I think the doctors should even negotiate with themselves. Now we have instituted that when a patient need DOTs [daily observed TB treatment], we [the public health nurses] go to the chest [clinic] with the doctors. So they [the doctors] will be able to speed up the treatment [initiation] for us. Initially we were going there alone but we have realised that NO, it takes a long time. So why do not we go there with the doctors, so that they will be able to champion the course for us…I mean for the patient.”… “Sometimes if you are lucky too, you will be given the medications. The moment the medication is being given to us, we can educate the patient to take it. As early as possible so every other morning they take it, before they take their meal.”* Public health nurse	

QT, Qualitative theme; HCW, healthcare worker; TB, Tuberculosis; LAM, lateral flow urine lipoarabinomannan assay, Determine™ TB LAM Ag test (Abbott Laboratories, Chicago, IL, USA, previously Alere); Xpert, Xpert = sputum Cepheid Xpert MTB/RIF assay or Xpert Ultra (Sunnyvale, California, United States).

#### 3.3.1 Barriers to routine sputum Xpert testing

A gap in access to routine TB testing using sputum Xpert was identified in the TBPOC study and explained in qualitative data by a lack of relatives to assist in sample logistics, negative beliefs around the disease, and patients being unable to produce a sputum sample.

#### 3.3.2 Barriers and facilitators to Determine LAM testing

The overall Determine LAM test procedure was experienced easily in the HCW survey, and almost all eligible patients in the TBPOC study received a test result. Reasons mentioned for not testing an eligible patient included that some patients were unwilling to take the test, which the HCWs ascribed to fear of or disbelief in modern medicine. One doctor explained that delayed HIV screening is a potential barrier to Determine LAM use.

#### 3.3.3 Timely TB diagnosis when assisted by Determine LAM compared to using Xpert only

The time from enrolment to TB diagnosis was reduced when Determine LAM was made available in addition to sputum Xpert in the TBPOC study. The HCW survey described that urine could be obtained on the same day as the request and that the Determine LAM procedure was estimated at a median of 30 min/patient. It was confirmed in the qualitative interviews that urine was easier to obtain than sputum. Delays in sputum-based TB testing as compared to Determine LAM testing were related to logistics with sample transportation and pick up of results, as well as Xpert laboratories being closed on the weekends.

#### 3.3.4 The place of Determine LAM testing in clinical practice was at the bedside

The TBPOC study and HCW survey demonstrated that almost all Determine LAM tests were performed at the bedside. The survey also revealed that more than half of HCWs would prefer performing the test in a laboratory or a dedicated room in the ward. It was understood from the qualitative data that nurses were concerned about patient confidentiality when testing at the bedside. However, doctors preferred bedside testing.

#### 3.3.5 Determine LAM test interpretation

Only 10% of respondents in the HCW survey did not use the reference scale card, and 10% thought it was challenging to interpret intensity bands. In the qualitative data, HCWs reported that it could be difficult to discriminate, especially between a grade 1 positive and a negative Determine LAM test result. Several HCWs described situations where they used more experienced colleagues to assist the Determine LAM procedure and interpretation.

#### 3.3.6 Barriers to Determine LAM guided TB treatment initiation

Quantitative findings from the TBPOC study identified that less than half of Determine LAM-positive patients initiated TB treatment. Qualitative data explained that the medical record was a weak link for bedside test result reporting. Others were concerned about the sub-optimal specificity.

#### 3.3.7 Routine healthcare practices compromised timely TB treatment initiation

The TBPOC study did not have any impact on the time to TB treatment initiation. Barriers identified included awaiting costly pre-treatment investigations, such as liver function tests. Moreover, HCWs reported that access to TB treatment was limited over the weekends and that TB medications by routine practice were only administered in early mornings.

## 4 Discussion

### 4.1 Discussion

This mixed methods study provides an understanding of how urine Determine LAM was received and implemented to diagnose and treat TB among PWH in a real-world Ghanaian in-hospital context.

We (15, 18) and others have previously shown that sputum-based TB investigation cannot stand alone, as only around half of PWH with presumptive TB obtain a test result (2, 7, 8). The mixed methods analysis explained this gap by patients being unable to produce a sputum sample, a main reason known from similar settings (1, 2, 28). HCWs expressed fear in handling sputum samples from patients with possible TB and experienced that negative beliefs around TB diagnosis among the patients could hamper sample collection, supported by another study from Ghana (29). Importantly for our setting, HCWs suggested that PWH often had limited socio-economic support with an impact on access to routine in-hospital healthcare.

Sample transportation to centralized Xpert laboratories is described to delay TB diagnosis (30). During the Determine LAM intervention, 93.1% of eligible patients received a urine Determine LAM test result (15). Both pre-test and disease counseling were regarded as important strategies to increase patient acceptance of TB testing and management. The most important facilitator for a same-day Determine LAM test was the ease of obtaining a urine sample compared to a sputum sample. Having the Determine LAM tests available at the POC empowered HCWs to realize rapid TB diagnosis and treatment, independent of the patient’s relatives and access to the TB laboratory. Among the methods recommended by the WHO to assist in identifying presumptive TB among PWH are W4SS, C-reactive protein >5 mg/L, chest X-ray, and molecular TB diagnostics, e.g., Xpert. However, in the setting of medical PWH inpatients, the W4SS, C-reactive protein, and chest X-ray have limited accuracy (extremely low specificity) (3). Using mWRD as an upfront screening and diagnostic test is warranted given the urgency of timely diagnosis in this population, and the suggested role of Determine LAM test is to assist in diagnosis (3). Up to now, Determine LAM is the only true POC TB diagnostic method accessible at the bedside with the advantage of reliance on urine, which is easily obtained compared to sputum.

The mixed methods analysis helped us understand the gap between having a positive Determine LAM test and initiating TB treatment. Barriers were often related to the local context, including limited access to TB medication on the weekends and difficulties obtaining liver and kidney function tests before treatment initiation. A feasibility study reported time to counseling and awaiting CD4 cell counts as barriers to Determine LAM-guided TB treatment initiation (11). In our study, HCWs did have access to CD4 cell count, but it was not a requirement for Determine LAM testing in the study context of inpatients or by WHO guidelines (4). Despite this, limitations in programmatic CD4 cell count testing were highlighted as a potential barrier to Determine LAM use by doctors in this study. We therefore suggest that current guidelines should be made more user-friendly and easily interpretable in clinical practice. Recent systematic reviews using individual patient data further suggest the Determine LAM testing in all HIV-positive medical inpatients be implemented (31, 32). A barrier to POC Determine LAM testing not previously described was delayed HIV screening of patients with unknown HIV status. The qualitative discussions further revealed a reluctance among HCWs to initiate TB treatment for Determine LAM-positive patients when pre-test TB suspicion was low due to concerns about false positive results. This discussion is also raised in other studies (10, 11). Our results finally echo that rapid treatment initiation can be delayed when not supported by a Determine LAM reporting system and 24-h access to TB medication (10).

Before this study, we did not question that the optimal use of Determine LAM was at the bedside. However, the mixed methods analysis revealed different opinions between staff categories, with nurses expressing concerns that bedside Determine LAM testing could compromise patient confidentiality. Ensuring patient confidentiality at the hospital ward may be challenging (33) and has not been sufficiently discussed regarding the Determine LAM test. A strategy used by HCWs in our setting was to use physical shields between patient beds.

Interpretation of the Determine LAM test was expected to be challenged by the use of the reference scale card and difficulties in discriminating between grade 1 positive and a negative test line, as bands of lower intensity than grade 1 may appear (10). Most HCWs in our study reported using the reference scale card, but one in 10 HCWs did not, which may lead to a misinterpretation of the test result. Similar interpretation issues have been reported from Mozambique and the Democratic Republic of the Congo (11), highlighting the importance of comprehensive HCW training in test interpretation. In our study, in line with others, HCWs often validated test results with assistance from a more experienced colleague (10).

### 4.2 Strengths and weaknesses

This real-world practice evaluation of the Determine LAM test’s role in diagnosing and guiding TB treatment used both rigorous quantitative and qualitative methods with integration at several levels. The quantitative findings may be generalized to similar secondary and tertiary health facilities admitting severely immunosuppressed PWH in Ghana and abroad. The qualitative analyses bring in-depth knowledge but are limited to discussions with HCWs at one large teaching hospital. Local practices and work culture may differ between tertiary health facilities in Ghana and other similar healthcare systems abroad, limiting the generalizability of the findings. Especially, the identified barriers to rapid turnaround time from sample collection to having an available test result, explained by the absence of relatives, and reduced access to TB investigations and medications on the weekends, related to in-hospital facilities’ limited opening hours, maybe specific for this setting. Future studies should include more diverse healthcare settings and patient interviews.

In the qualitative analyses, we obtained insights into the study context, the Determine LAM user perspectives, and the Determine LAM contextual role in clinical practice. Using pre-defined codes in the qualitative analysis may lead the researcher to find support rather than non-support of their data and cause bias (22). Contrarily, the pre-determined codes ensured that important findings related to previous research in the field and key findings from the quantitative methods were not missed during the analysis. Furthermore, we allowed new codes to emerge and based the emerging overarching themes on pre-defined and new codes, limiting the risk of bias.

Since a non-Ghanaian investigator (J.Å.) moderated the qualitative interviews and conducted the primary analysis, this could potentially have led to misunderstandings or oversights. To minimize this risk, the moderator regularly summarized the discussions to confirm understanding with the participants during the interviews. A Ghanaian research assistant with a good understanding of the context was present during the interviews. Several Ghanaian and Danish investigators with previous experience with the topic, the context, and the research methodology were part of the design of the study and the revision of the original manuscript draft.

There is also a risk that the interviewees felt they had to speak positively about the Determine LAM test, as the moderator was the study manager for the previous intervention study. To prevent this, we tried to facilitate a free discussion by starting each interview by explaining that there were no right or wrong answers and that the aim was to learn how the interviewee experienced this as their different opinions and perspectives would be helpful to critically assess the appropriateness of the Determine LAM test.

The integration of quantitative and qualitative methods enabled us to better understand the reasons for the identified gaps described in the quantitative methods and how the Determine LAM intervention was received and worked in a Ghanaian in-hospital context, increasing the overall value of the respective methods.

### 4.3 Conclusion

Determine LAM testing was feasible among inpatients with HIV at three hospitals in Ghana and empowered HCWs in timely TB diagnosis. We highlight different views on the best location for Determine LAM testing and suggest that future research should explore the patient experiences and perspectives of bedside testing and disclosure of test results. Identified gaps in the routine and the Determine LAM-enhanced in-hospital TB care cascade were explained by the mixed methods analysis. These barriers were often related to the context, such as structural barriers to TB investigation and treatment initiation. Our study may assist in improving the in-hospital quality of care and may guide clinical Determine LAM scale-up in similar settings.

## Data availability statement

The study participant’s rights and identities are protected, therefore the raw data such as interview transcripts are not available. Requests regarding the datasets should be directed to the first author JÅ.

## Ethics statement

The studies involving humans were approved by Ghana Health Service Ethics Review Committee for research conducted at TGH and LH (GHS-ERC 006/06/19, August 19, 2019) and by the Scientific and Technical Committee and the Institutional Review Committee for research conducted at KBTH (KBTH-IRB/00052/2019, September 9, 2019). The studies were conducted in accordance with the local legislation and institutional requirements. The participants provided their written informed consent to participate in this study.

## Author contributions

JÅ: Conceptualization, Data curation, Formal analysis, Funding acquisition, Investigation, Methodology, Project administration, Resources, Software, Validation, Visualization, Writing – original draft, Writing – review & editing. BT: Data curation, Formal analysis, Investigation, Methodology, Validation, Writing – review & editing. PP: Data curation, Investigation, Writing – review & editing. AK: Data curation, Investigation, Writing – review & editing. JC: Data curation, Investigation, Writing – review & editing. YA-P: Data curation, Investigation, Writing – review & editing. EM: Formal analysis, Investigation, Methodology, Validation, Writing – review & editing. ÅA: Investigation, Supervision, Writing – review & editing. EK: Investigation, Supervision, Writing – review & editing. ML: Conceptualization, Investigation, Supervision, Writing – review & editing. IJ: Conceptualization, Funding acquisition, Investigation, Project administration, Resources, Supervision, Writing – review & editing. SB: Conceptualization, Data curation, Formal analysis, Investigation, Methodology, Project administration, Supervision, Validation, Writing – review & editing.

## Footnotes

^1^ClinicalTrials.gov: NCT04122404.
