# Supplementary material for: Use of the urine Determine LAM test in the context of tuberculosis diagnosis among inpatients with HIV in Ghana: a mixed methods study
Source: Front Public Health. 2024 Jan 5;11:1271763. doi: 10.3389/fpubh.2023.1271763 (PMC10797072; doi:10.3389/fpubh.2023.1271763)
Supplement: Supplementary file 1 [file Data_Sheet_1.PDF]

frontiers

Supplementary Material

Article Title

Use of the urine Determine LAM test in the context of TB diagnosis among inpatients with HIV in Ghana – a mixed methods study

Johanna Åhsberg[1,2,3*], Britt Pinkowski Tersbøl[4], Peter Puplampu[5], Augustine Kwashie[6], Joseph Oliver Commey[7], Yaw Adusi-Poku[8], Ellen Moseholm[9,10], Åse Bengård Andersen[11], Ernest Kenu[12], Margaret Lartey[5], Isik Somuncu Johansen[1,2], Stephanie Bjerrum[1,11]

[1]Research Center of Infectious Diseases, Department of Clinical Research, University of Southern Denmark, Odense, Denmark

[2]Mycobacterial Centre for Research Southern Denmark, MyCRESD, Department of Infectious Diseases, Odense University Hospital Odense, Denmark

[3]International Reference Laboratory of Mycobacteriology, Statens Serum Institut, Copenhagen, Denmark.

[4]Global Health Section, Department of Public Health, University of Copenhagen, Copenhagen, Denmark

[5]Department of Medicine & Therapeutics, Medical school, College of Health Sciences, University of Ghana, Accra, Ghana

[6]Department of Medicine, Tema General Hospital, Tema, Ghana

[7]Department of Medicine, Lekma Hospital, Teshie, Ghana

[8]National Tuberculosis Control Programme, Ghana Health Service, Korle-Bu, Accra, Ghana

[9]Department of Infectious Diseases, Copenhagen University Hospital, Hvidovre

[10]Department of Public Health, Faculty of Health and Medical Sciences, University of Copenhagen, Copenhagen, Denmark

[11]Department of Infectious Diseases, Copenhagen University Hospital Rigshospitalet, Copenhagen, Denmark

[12]Department of Epidemiology and Disease Control, University of Ghana, Accra, Ghana

*** Correspondence: Johanna Åhsberg**
johanna.maria.aahsberg@rsyd.dk

1 Supplementary Tables

Table S1. Focus group discussion and In-depth interview topic guides

**Topic guide for Focus group discussion among clinical staff**	
**TBPOC study**	
**Date:**	
**Location:**	
**Objective**	Evaluation of values and preferences for the implementation of Determine LAM (Abbott) urine test for TB
**Participant´s profile**	Healthcare provider attending FGD:
**Research staff and roles**	
**Intro**	Thank you for joining our focus group discussion today<br>We are going to talk about diagnosis of tuberculosis<br>We are here to hear about your experiences with use of urine LAM<br>The session will last about 60 min
**Consent**	1. Participation<br>2. Recording<br>3. Publication, anonymized
**Discussion**	No right or wrong answers; we want to learn from you<br>Be honest<br>We will try to let everyone talk<br>Raise a hand
**Warm up**	(*Show timeline from "asymptomatic patient" to "on TB treatment"*)<br><br>Look at the timeline for diagnosing TB among admitted PLHIV and help me understand the different steps where you are active in your role as a healthcare worker
**Discussion**<br><br>*Possible short intro to LAM and target group (PLHIV), and WHO guidelines*	**Main discussion point:**<br>From your experience with working with TB what are your opinions on the use of LAM among PLHIV?<br><br>**Sub-questions (follow up):**<br>1: What are the benefits of using the LAM test?

Introduction to the possible national roll out.	2: How do you think the LAM test would benefit the individual PLHIV? (***prompt*** *for specific experiences using the LAM test*)<br><br>3: What are the challenges with using the LAM? (***prompt*** *for specific experiences using the LAM test*)<br><br>4: In your opinion, where is the optimal place to perform the LAM test?<br><br>5: Who do you think should refer to, perform and interpret the LAM test?<br><br>6: What are your recommendations to the control programmes if they decide to roll out the LAM test nationally?
**Conclusion**	Additions from anyone something they want to add?<br>Recap main findings to confirm understanding.<br>Thank you very much for your help.

**Interview guide for semi-structured In-depth interviews of clinical staff**	
**TBPOC study**	
**Date:**	
**Location:**	
**Objective:**	Evaluation of values and preferences for the implementation of Determine LAM (Abbott) urine test for TB
**Participant´s profile:**	
**Research staff:**	
**Intro:**	Thank you for participating in the interview
	We are going to talk about diagnosis of tuberculosis
	We are here to hear about your experiences with the use of urine LAM

	The session will last about 50 min	
**Consent:**	1. Participation	
	2. Recording	
	3. Publication, anonymized	
**Discussion:**	No right or wrong answers; we want to learn from you; be honest, comfortable	
**Interview**		
	**Discussion point**	**Questions**
Show timeline from "asymptomatic patient" to "on TB treatment"	Routine TB cascade among PLHIV	**1.** Look at the timeline for diagnosing TB among admitted PLHIV and help me to understand the different steps where you are active.
		**2.** How would you describe the typical process from admission to TB treatment among PLHIV.
		Prompt: *Which steps in the TB timeline works well in your workplace? Can you describe any steps in the TB timeline that are challenging? How can these steps be improved?*
	**Main discussion point**	**Questions**
Possible short intro to LAM and WHO guidelines available	LAM test user perspectives	1. From your experience with working with TB what are your opinions on using the LAM test?
		2. From you experience with using the LAM test, what are the benefits?
		3. How do you think the LAM test benefit the individual PLHIV?

		Prompt: *If possible, please describe a situation where you have experienced that a patient benefitted from the LAM test.*
		4. What are the challenges with using the LAM test?
		Prompt: *Screening for eligibility and referring to the test; Sampling of urine; SOP/test manual; Testing location, Person that performs the test; Test procedure; Test turnaround time; Reading the result using the reference scale card; Interpretation as positive or negative; Guide TB treatment initiation*
		Prompt: *If possible, can you describe a situation with a patient where you have experienced challenges with using the LAM test?*
		5. In your opinion, where is the optimal place to perform the LAM test?
		6. Who do you think should refer to, perform and interpret the LAM test?
Introduction to the possible national roll out.		7. Help me understand which steps in the LAM procedure that you would need to learn more about, if any?
		8. Which type of training would be most helpful for learning the test procedure?
		7. What are your recommendations to the control programmes if they decide to roll out the LAM test nationally?
**End of the Interview**		
		What did you think about this interview? Anything you think I should have asked you about?
		Recap main findings to confirm understanding.
		Thank you very much for your help.

Table S2. Characteristics of healthcare workers responding to the quantitative staff survey on Determine LAM feasibility and ease of use, overall and stratified by hospital, n=81

		**Overall population (n=81)**	**LH (n=25)**	**KBTH (n=44)**	**TGH (n=12)**
Staff category	Medical doctor	28 (34.6)	9 (36.0)	16 (36.3)	3 (25.0)
	Nurse	42 (51.9)	16 (64.0)	23 (52.3)	3 (25.0)
	Clinical officer or Physician assistant*	3 (3.7)	0 (0.0)	0 (0.0)	3 (25.0)
	Counsellor, Health assistant or Volunteer*	5 (6.2)	0 (0.0)	2 (4.5)	3 (25.0)
	TB diagnostic staff	3 (3.7)	0 (0.0)	3 (6.8)	0 (0.0)
Work experience	<1 year	22 (27.2)	3 (12.0)	14 (31.8)	5 (41.7)
	1-3 years	20 (24.7)	3 (12.0)	13 (29.6)	4 (33.3)
	3-5 years	9 (11.1)	5 (20.0)	3 (6.8)	1 (8.3)
	>5 years	30 (37.0)	14 (56.0)	14 (31.8)	2 (16.7)
Role in TB diagnosis**	Research or Administration and management*	7 (8.6)	1 (4.0)	5 (11.4)	1 (8.3)
	Clinical management of patients	65 (80.2)	24 (96.0)	35 (79.5)	6 (50.0)
	TB screening or diagnosing TB in the laboratory*	5 (6.2)	0 (0)	3 (6.8)	2 (16.7)
	Health education among patients or Volunteering*	4 (4.9)	0 (0)	2 (4.5)	2 (16.7)
	Service learning or being a Student*	3 (3.7)	0 (0)	2 (4.5)	1 (8.3)
How many patients do you refer for TB investigation each week?	0-1 patient	23 (28.4)	5 (20.0)	13 (29.6)	5 (41.7)
	2-4 patients	38 (46.9)	17 (68.0)	15 (34.1)	6 (50.0)
	5-7 patients	7 (8.6)	2 (8.0)	4 (9.1)	1 (8.3)
	> 7 patients	6 (7.4)	1 (4.0)	5 (11.4)	0 (0)
	NA	7 (8.6)	0 (0)	7 (8.6)	0 (0)
How many patients do you test with	0-1 patients	37 (46.3)	9 (37.5)	22 (50.0)	6 (50.0)
	2-4 patients	26 (32.5)	13 (54.2)	8 (18.2)	5 (41.7)
	5-7 patients	2 (2.5)	1 (4.2)	0 (0)	1 (8.3)

Determine LAM per week?	> 7 patients	0 (0)	0 (0)	0 (0)	0 (0)
	NA	15 (18.8)	1 (4.2)	14 (31.8)	0 (0)

Abbreviations: LH=Lekma Hospital; KBTH=Korle Bu Teaching Hospital; TGH=Tema General Hospital; TB=Tuberculosis; Determine LAM=lateral flow urine lipoarabinomannan assay, Determine DetermineTM TB LAM Ag test (Abbott Laboratories, Chicago, IL, USA); NA=Not applicable

Data are n (%).

* Categories with less than 3 responders were merged with 1-2 other categories.

** More than one alternative allowed per respondent.

Missing values were excluded from analysis. Number of participants with missing values per survey question: How many patients do you test with Determine LAM per week (1)
